# Failures in Bridge-Work

**Published:** 1899-09

**Authors:** F. M. Willis

**Affiliations:** Ithaca, N. Y.


ARTICLE V.

### FAILURES IN BRIDGE-WORK.

DR. F. M. WILLIS, ITHACA, N. Y.

This will undoubtedly appeal to every dentist, because we have all seen failures in bridge work. Some of us have made failures, and to such persons anything said in favor of bridging will be of unusual interest. A great deal has been said and written recently which has been adverse to this branch of dentistry, and perhaps a few words from one who is making bridge work a life study might encourage some disheartened one who is ready to give up such work on account of a few failures.

The dentist who saves teeth is the greatest benefactor to the human race, and next to him is the one who can supply lost teeth by the most useful and comfortable method. Bridge work fills a place in dentistry which can never be taken by anything else. A properly constructed bridge on suitable anchorages is the most useful, comfortable of all artificial substitutes. Why have there been so many failures in this particular line of work ? I shall endeavor to point out some of the reasons.

In the first place, the construction of bridges has been attempted by men who have not been properly instructed in this line of work. Until recently the colleges have not given sufficient instruction in bridge work, and so the college graduate has attempted to do something which he did not understand, and the result has been failure. Then, too, a great many who were already in the profession when bridge work was first introduced, have attempted to do this work, having had but little, if any, instruction, and the natural result has been failure. To be a successful bridge worker requires a thorough knowledge of working metals, intelligent instruction in this particular line of work and a large share of practical experience.

In the second place, failures have resulted from using unhealthy teeth or roots for abutments, or from attaching too large a bridge to too few anchorages. Experience has proven that the most successful bridges have been small ones which were attached to strong, healthy teeth or roots. Large bridges of ten, twelve or fourteen teeth are seldom admissible under any circumstances. Sometimes it is safe to supply an entire upper or lower denture by three or four small bridges, but it is very seldom wise to construct a permanent bridge of more than six or eight teeth in one piece.

Another cause of failure is the lack of knowledge in the construction of the Richmond crown. For a single crown it is sometimes possible to get along without banding the root, but as a supporter for bridge work nothing short of a properly constructed Richmond crown will serve for the anchorage to any of the anterior teeth. A properly constructed Richmond means a healthy, well-filled root, the end of which must be reduced to the inverted cone shape, a delicate, well-fitting band under the gum, a strong platino-iridium post in the root and a snug fitting porcelain, the cutting edge of which is sufficiently protected by gold so that it will not break in mastication. This protection can be obtained in such a way as not to show any gold, and yet absolutely prevent any danger of subsequent breakage from mastication.

Other failures have resulted from ill-fitting bands in making the anchorages. The edge of the band near or under the gum must fit absolutely. If the tooth cannot be trimmed so that the band will fit closely under the gum, then it will be better not to extend the band beyond that portion of the tooth where it does fit closely. It is seldom absolutely necessary that the band on any of the posterior teeth should extend under the gum, so that any future decay may be properly cared for. An ill-fitting band that extends under the gum hastens decay rather than prevents it, and in such a case the decay is not likely to be discovered until the anchorage is almost, or entirely destroyed. No artificial crown will prevent decay unless it is an absolute fit and unless the tooth or root can be kept perfectly dry until the crown is cemented.

Another difficulty arises from too much grinding of teeth with live pulps. A live tooth will always last longer than one with a dead pulp, but a live pulp has often been destroyed by entirely denuding the tooth of enamel, in order that the band might fit well under the gum. A live tooth should be ground as little as possible.

Many times bridge work fails because it is not made strong enough. It has been said that in constructing bicycles, the strain which the machines will be required to stand is carefully computed, and then they are made so that they will withstand five times that strain. The same rule should be adopted in bridge work; make them five times stronger than seems necessary.

The occlusion with the teeth in the opposite jaw has a great deal to do with the success of bridge work. Nothing short of perfection in particular will answer. A faulty articulation will often destroy what might otherwise be a permanent and satisfactory piece of work.

Any cavities, or even slight decay, about the anchorages should be carefully filled before the bridge is cemented. It is also a wise safeguard to dry the teeth or roots carefully, coat them with nitrate of silver and then drive off all moisture with a hot air blast before cementing any bridge. If the anchorages are Richmond crowns, it is well that the cementing be done as suggested by Dr. Evans—by coating the posts with chloro-percha and trying on, then cement in the usual way. By applying a warm instrument to such a bridge for five minutes the gutta percha will soften and the piece can be removed.

The last reason for so many failures is the greatest reason of all. Dentists who are not skilful enough to construct their own bridges will take an impression of the case and send a plaster model to some of the numerous laboratories where bridges are constructed with neatness and despatch, and expect the result to be satisfactory.

Bridge work done in this way will rarely be successful, and such procedure cannot be too strongly condemned. No man ought to attempt bridge work who cnnnot do the work himself, and begin by fitting the bands to the anchorages in the mouth. Bands fitted to plaster models may succeed in some cases, but no method is so satisfactory as to fit the bands to the teeth or roots in the mouth.

Patients will often ask how long bridge work will last. I used to reply, "It will last a lifetime;" now I reply, "It will probably last several years." I find by experience that the latter statement is safer. A bridge which gives the patient good service for five, eight or ten years should not be considered a failure. In fact, I find many patients who are willing to endure the discomfort and expense of having bridge work done, if they can be assured that it will last five years.

In skilful and conscientious hands, bridge work is not a failure. On the other hand, a great many patients who are wearing clean, strong, useful bridges are willing to testify to the fact that bridge work is a success.—*Items of Interest.*

